# Complex antidepressant therapy with the inclusion of various neuroprotectors in inpatient gerontopsychiatric practice

**DOI:** 10.1192/j.eurpsy.2024.495

**Published:** 2024-08-27

**Authors:** V. Pochueva, T. Safarova, O. Yakovleva

**Affiliations:** FSBSI “Mental Health Research Center”, Moscow, Russian Federation

## Abstract

**Introduction:**

Depressions are the most common mental disorders in elderly and senile patients. In these patients, there is a decrease in neurotrophic potential. Treatment of such patients with antidepressants alone does not always allow to achieve complete normalization of the secretion of neurotrophic factors and complete restoration of neurogenesis processes. In this regard, it is important to expand therapeutic opportunities to develop new therapeutic strategies for pharmacotherapy of late-age depression.

**Objectives:**

Comparative evaluation of the effectiveness of two types of complex antidepressant therapy with the inclusion of different neuroprotectors (actovegin or cerebrolysin) in the treatment of late-life depression in the therapeutic regimen.

**Methods:**

The study included 2 groups of patients with mild and moderate depressive episode (DE), comparable in basic demographic and clinical parameters.

The 1st group included 21 people, including 7 men (33.3%) and 14 women (66.7%), median age were 69 years [66; 76]. In 10 patients (47.6%), DE was diagnosed as part of recurrent depressive disorder (DDR), in 9 patients (42.9%) - as part of bipolar affective disorder (BAR), and in 2 patients (9.5%) is a single DE. Group 2 included 20 patients, 5 of them men (25%) and 15 women (75%), median age were 64 years [62; 70]. In 11 patients (55%), DE was diagnosed as part of DDR, in 6 patients (30%) - as part of BAR, and in 3 patients (15%) – single DE.

The 1st group of patients received complex antidepressant therapy with the inclusion of actovegin for one month, the 2nd group - with the inclusion of cerebrolysin. The effectiveness of the therapy was assessed on the HAMD-17 and HARS scales.

**Results:**

A comparative study demonstrated the effectiveness of both types of complex antidepressant therapy used.

A comparative assessment of the effectiveness of the therapeutic response in two groups of patients showed no statistically significant differences in the reduction of depressive disorders after 2 weeks of therapy. Only by the end of the therapeutic course there was a more pronounced reduction of depressive disorders in the 1st therapeutic group (73.6% vs 63.6% (p < 0.05)).

Reduction of anxiety disorders, assessed on the HARS scale, was noted both by the 14th and 28th day of therapy in both therapeutic groups. However, it turned out to be more pronounced in the 1st therapeutic group: by the 14th day of therapy, the reduction of anxiety in the 1st and 2nd groups of patients was 36.4% and 30.0%, respectively (p < 0.05), and by the end of therapy - 77.7% and 60.0%, respectively (p < 0.01).

**Conclusions:**

The augmentation of antidepressant therapy with drugs with multimodal activity, actovegin and cerebrolysin, should be considered as effective and it can be recommended for inclusion in the therapeutic regimen for the treatment of late-age depression in a psychiatric hospital.

**Disclosure of Interest:**

None Declared

